# Serum Resistin and Insulin-Like Growth Factor-1 Levels in Patients with Hypothyroidism and Hyperthyroidism

**DOI:** 10.1155/2013/306750

**Published:** 2013-02-24

**Authors:** Ceren Eke Koyuncu, Sembol Turkmen Yildirmak, Mustafa Temizel, Tevfik Ozpacaci, Pinar Gunel, Mustafa Cakmak, Yüksel Gülen Ozbanazi

**Affiliations:** ^1^Department of Clinical Biochemistry, Ministry of Health Okmeydani Educational and Research Hospital, Okmeydani, Istanbul 34384, Turkey; ^2^Department of Internal Medicine, Ministry of Health Okmeydani Educational and Research Hospital, Okmeydani, Istanbul 34384, Turkey; ^3^Department of Nuclear Medicine, Ministry of Health Okmeydani Educational and Research Hospital, Okmeydani, Istanbul 34384, Turkey; ^4^Department of Biostatistics, Uludag University Medical Faculty, Bursa 16059, Turkey; ^5^Department of Clinical Chemistry, Gulkent State Hospital, Isparta 32100, Turkey

## Abstract

*Introduction*. The aim of this study was to evaluate the serum levels of resistin and insulin-like growth factor-1 (IGF-1) and and also the potential relationship between thyroid function and levels of resistin and IGF-1 in hypothyroid and hyperthyroid patients. *Methods*. Fifteen cases of hypothyroid (HT), 16 of subclinically hypothyroid (SCHT), 15 of hyperthyroid (HrT), 15 of subclinically hyperthyroid (SCHrT), and 17 healthy individuals have been included in the study. Serum resistin levels were measured using enzyme-linked immunosorbent assay and IGF-1 and thyroid stimulating hormone (TSH) levels by chemiluminescence method. *Results*. Resistin levels in total HT group were significantly higher than in controls (12.66 ± 6.04 and 8.45 ± 2.90 ng/mL, resp.). In SCHrT subgroup resistin levels were significantly higher than those of controls (14.88 ± 7.73 and 8.45 ± 2.90 ng/mL, resp.). IGF-1 levels were significantly lower in total HT than in total HrT and control groups (117.22 ± 52.03, 155.17 ± 51.67, and 184.00 ± 49.73 ng/mL, resp.). Furthermore IGF-1 levels in HT subgroup were significantly lower compared to controls (123.70 ± 44.03 and 184 ± 49.73 ng/mL, resp.). In SCHT subgroup IGF-1 levels were significantly lower than those of control and SCHrT groups (111.11 ± 59.35, 184.00 ± 49.73, and 166.60 ± 47.87 ng/mL, resp.). There were significant correlations between IGF-1 and TSH in HT subgroup and between resistin and TSH in total HrT group. *Conclusion*. It was concluded that increased resistin levels are directly related to thyroid dysfunction, and GH/IGF-1 axis is influenced in clinically or subclinically hypothyroidism patients.

## 1. Introduction

Thyroxine (T4) and triiodothyronine (T3) hormones regulate heat production and energy utilization, and they are very important for normal growth and development. They also play important roles in regulating various homeostatic mechanisms [1]. Thyroid abnormalities are accompanied by changes in intermediary metabolism including alterations in body weight, insulin resistance, and lipid profile [2].

It has been understood that adipose tissue is not only a passive energy reservoir, it is but also an active endocrine tissue. Resistin is an adipocytokine which is considered to have an important role in energy metabolism and is secreted from adipocytes and macrophages. Resistin antagonizes insulin effect and causes insulin resistance especially in obese patients. Resistin is also playing a role in inflammation and is a potential biomarker in cardiovascular and many other diseases [3, 4].

IGF-1 is a growth factor which is secreted from liver as a response to growth hormone (GH). IGF-1 has many receptors in various tissues and manifests insulin-like effect as well; it functions in many metabolic pathways including energy metabolism [5]. There are very few studies concerning resistin and IGF-1 concentrations in patients with thyroid dysfunction, and they present conflicting results [4, 6–9]. Furthermore there is no study in the literature related to correlation between resistin and IGF-1 in thyroid dysfunction.

The aims of the present study were (1) to determine serum resistin, IGF-1, TSH, fT4, lipid, and transaminase levels together in untreated patients with hyper-and hypothyroidism and control groups for the first time and (2) to investigate potential relationship between thyroid function and serum levels of resistin and IGF-1 in HT and HrT patients.

## 2. Materials and Methods

The study consisted of 15 HT patients with ages 20–76, 16 SCHT patients with ages 26–71, 15 HrT patients with ages 22–79, 15 SCHrT patients with ages 26–70, and control group consisting of 17 individuals who were detected as healthy by physical examination and laboratory findings. The total number of volunteers was 78. 

The individuals having TSH levels over 5.6 *μ*IU/mL and serum free T4 (fT4) levels below 0.58 ng/dL were classified as HT; the individuals having TSH levels over 5.6 *μ*IU/mL and serum fT4 levels 0.58–1.64 ng/dL were classified as SCHT. 

The individuals having TSH levels below 0.34 *μ*IU/mL and serum fT4 levels over 1.64 ng/dL were classified as HrT; the individuals having TSH levels below 0.34 *μ*IU/mL and serum fT4 levels 0.58–1.64 ng/dL were classified as SCHrT. The patients who have TSH levels over 5.6 *μ*IU/mL were classified as total HT, and the patients who have TSH levels below 0.34 *μ*IU/mL were classified as total HrT.

In patient groups there was no individual who had any systemic disease. None of the patients had treatment concerning thyroid dysfunction.

Detailed histories of individuals included in the study were obtained; follow-up forms were prepared. All of the tests and clinical meanings of the tests performed were explained to the individuals, and their written consents were obtained. The ethical approval of study was given by the Okmeydani Educational and Research Hospital Ethics Committee.

10 mL of blood samples into plain vacuum tubes with gel were obtained after 12 hour fasting. They were centrifuged for 10 min at 2000 ×g. T-Chol, HDL-Chol, AST, ALT, TSH, and fT4 were analyzed on the same day. Undiluted serum samples were seperated into two tubes, and they were stored at −80°C for maximum 6 months. Repetative freezing and thawing were not performed. 

Serum T-Chol, HDL-Chol, AST, and ALT levels were determined photometrically in Beckman Coulter AU2700 auto-analyzer (Beckman Coulter Inc., CA, USA); TSH ve fT4 levels were determined in Beckman Coulter UniCel DxI 800 autoanalyzer (Beckman Coulter Inc., CA, USA) by using access high sensitive TSH 3rd generation and access fT4 successively by chemiluminescence method. LDL-Chol levels were calculated by the Friedewald formula. AdipoGen Human Resistin ELISA kit (AdipoGen Inc., Incheon, Korea) was used to determine resistin levels. IGF-1 levels were determined in Siemens Immulite 2000 analyzer (Siemens Healthcare Diagnostics, Deerfield, IL, USA) by solid phase enzyme marked chemiluminescence method.

In order to analyze data SPSS 17.0 ve GraphPad InStat 3.05 packet programs were used. Standart deviations (SD) and means of the Gaussian parameters included in the study were given in terms of groups. Both mean and min-max values and mean ± SD values were presented for the nonGaussian parameters. In comparison of more than two groups that were independent of each other (3 groups and 5 groups), Kruskal-Wallis and one way ANOVA tests were used. The groups which were determined to be different from one another in terms of parameters were compared again in binary groups. The correlation between resistin, IGF-I, and TSH was presented via Spearman's correlation coefficient. The results were evaluated in 95% confidence and significance in *P* < 0.05.

## 3. Results

Of the individuals included in the study were 17 (22%) males and 61 (78%) females. Laboratory findings and demographic properties of total HT, total HrT, and healthy control groups and HT, SCHT, HrT, and SCHrT subgroups were showed in Tables 1 and 2, respectively. A significant positive correlation between resistin and TSH in total HrT group was detected (*r* = 0.463, *P* = 0.010) (Table 3). There was a statistically significant negative correlation between IGF-1 and TSH in HT subgroup (*r*  =  −0.704, *P* = 0.003) (Table 3). Within and between assay variations of resistin and IGF-1 measurements were shown at Table 4.

## 4. Discussion

Significantly higher T-Chol and LDL-Chol levels in total HT group were observed in comparison to total HrT and control groups. Significantly higher T-Chol and LDL-Chol levels in HT subgroup were observed in comparison to HrT, SCHrT, and control groups. These parameters were also significantly higher in SCHT in comparison to HrT subgroup. These findings support the possible correlation between thyroid function and lipid metabolism. 

Serum AST levels in total HT and HT groups were detected as significantly higher in comparison to control group. Hepatocellular damage due to fatty liver caused by hypercholesterolemia in hypothyroid patients causes high AST levels. Our findings are consistent with this situation.

Thyroid gland and adipose tissue functions are closely correlated with each other. Recently by the discovery of endocrine functions of adipose tissue, it has been considered that adipose tissue not only has storage function but also has active regulatory functions in energy metabolism [10]. Resistin which is one of the hormones secreted from adipose tissue is responsible for insulin resistance, and it is a candidate marker for insulin resistance. However the correlations between resistin and thyroid hormones which are among the major regulators in energy metabolism could not be clearly established.

Changes in thyroid hormone levels have been clearly known to affect insulin secretion and sensitivity. Correlation between resistin and thyroid functions have been investigated by both human and animal studies. Syed et al. [11] reported they observed significant improvement in insulin resistance in obese rats after exogenous thyroid hormone treatment. Nogueiras et al. [6] observed resistin mRNA levels increased in adipose tissue in hypothyroid rats, whereas it decreased to almost undetectable levels in hyperthyroid rats. 

In order to enlighten functions of resistin in human it is neccessary to accomplish detailed studies. There is very little information about relationship between thyroid functions and resistin. Furthermore existing studies present conflicting results [4, 6–9].

Significantly higher resistin levels in total HT and total patient group were observed in comparison to control group. It was also observed serum resistin level of total HrT group was higher in comparison to control group. But that elevation was not statistically significant. Serum resistin levels of SCHrT subgroup were significantly higher than those of control group. Although they are not statistically significant, the means of resistin levels in HT, SCHT, and HrT subgroups were higher than those of control group. 

The study of Yaturu et al. [10] consisted of 69 Graves' patients as hyperthyroid group and 32 of them taking radioactive iodine as hypothyroid group. Resistin levels of patients who are in hyperthyroid status were determined higher, when compared to the ones who are in hypothyroid status, and it was positively correlated to fT4 and fT3 and negatively correlated to TSH.

In the study of Krassas et al. [12] hyperthyroid group consisting of 43 patients was compared with both 23 healthy controls and 36 treated euthyroid patients who were previously hyperthyroid. Serum resistin levels of hyperthyroid patient group were higher than those of control group. However after normalization of thyroid hormones in hyperthyroid patients, they decreased, and it was correlated with control group. 

In another study consisting of 53 hypothyroid patients and 30 controls, Krassas et al. [9] also did not observe any difference between control and patient groups in terms of resistin. After a 4-5-month treatment, normalization of thyroid hormone levels did not result in a signficant change in resistin levels. Furthermore, after treatment, there was no difference in terms of resistin levels in euthyroid individuals. Resistin levels, thyroid hormones, TSH, thyroid antibodies, insulin levels, HOMA-IR index, and age, before treatment and after treatment, did not manifest correlation [9].

In their study consisting of 20 hypothyroid patients and 20 healthy controls, Iglesias et al. [7] observed similar resistin levels in pretreament and posttreatment period in hypothyroid patients, whereas those levels were significantly lower in hypothyroid patients than euthyroid control group [7]. Same research group in their study consisting of 20 hyperthyroid and 20 controls observed lower resistin levels in hyperthyroid patient group in comparison to control group. After normalization of thyroid function in hyperthyroid patients, they did not observe any significant change in resistin levels [7].

In this study higher resistin levels in total hypothyroid group conflict in comparison to control group with study of Krassas et al. [9] in which they did not observe any difference and with study of Iglesias et al. [7] in which they observed lower resistin levels. This contradiction may be as a result of inequal demographic characteristics and therapeutic variations of patient group. Although observed higher resistin levels were in total HT group in comparison to control group, no correlation between resistin and TSH in total HT group was found. This finding is consistent with that of Krassas et al. [9]. There were no proportional correlations between resistin levels and severity of hypothyroidism, whereas the total HrT group showed positive correlation between resistin and TSH levels. This finding conflicts with study of Yaturu et al. [10] in which they observed negative correlation. In this study, resistin levels were found higher in SCHrT subgroup than in controls. This finding is consistent with study of Krassas et al. [12] in which serum resistin levels of HrT patient group were higher than those of control group and conflict with Iglesias et al. [7] in which serum resistin levels of HrT patient group were lower than those of control group.

Consequently, when limited number of studies about resistin examined, it was observed that resistin levels are variable in thyroid dysfunctions [9, 10, 12]. Higher resistin levels in both hypothyroidism and hyperthyroidism were observed compared to controls. These findings refer to a relationship between thyroid dysfunction and serum resistin levels. Conflicting results may be related to secretion of resistin by macrophages in addition to adipose tissue in humans. Differences in inflammation status of selected individuals may cause different results. In this study, individuals were classified according to their serum levels of TSH, and fT4 and it was not known what was underlying the disease of hypo-and hyperthyroid patients. This is the limitation of this study.

Before now, the correlation between growth hormone excess, insulin resistance, and carbohydrate intolerance has been presented by some studies [13–16]. It has been considered that the correlation between insulin resistances is due to both growth hormone's insulin-like effect and its structural similarity to insulin causing activation of IGF-1 by binding weakly to insulin receptors. In acromegalic patients there is excess of growth hormone and consequently IGF-1 excess, and this results in glucose intolerance and type II diabetes. Besides, in a study performed by Silha et al., similar resistin levels in control subjects and acromegalic patients having high IGF-1 levels were observed [13]. They reported that insulin resistance in acromegaly may be due to other reasons than resistin [13]. In a study performed with transgenic GH-suppressed rats, Chiba et al. noted that low IGF-1 levels decrease resistin gene expression in white adipose tissue and plasma resistin levels, and insulin efficiency increases in dwarf rats [17]. Chen et al. reported IGF-1 decreases resistin gene expression and protein secretion in vitro [18]. Willemsen et al. performed a 2-year study with infants having low birth weight and short stature according to gestational age [19]. During this period they determined resistin levels in children having high IGF-1 levels and taking GH treatment. They observed resistin levels did not decrease after treatment, but levels of resistin were lower in children with high IGF-1 in comparison to control group. However they also observed that resistin levels increased spontaneously in children taking no treatment after 2 years, and there was a correlation between high IGF-1 levels and low resistin levels [19]. 

In another study performed with children having GH deficiency, Nozue et al. investigated effect of GH treatment on serum resistin levels [20]. In contrast to the study performed by Willemsen et al. [19], they reported that IGF-1 and resistin levels increased significantly after treatment with GH [20]. Schmid et al. detected that T4 replacement increased IGF-1 level in primary and central hypothyroid patients [21]. They reported that administration of thyroid hormone to the patients with GH deficiency increased IGF-1 level [21]. Völzke et al. detected high IGF-1 levels were correlated with goitre in males with presence of thyroid nodules and in females with low TSH levels [22]. Kursunluoglu et al. reported IGF-1 polymorphism can be a risk factor for hypothyroidism [23]. Akin et al. observed significantly lower serum IGF-1 levels in subclinically hypothyroid patients in comparison to control group, whereas they observed similar IGF-1 levels in subclinically hyperthyroid patients with control group [24]. They reported GH/IGF-1 axis was influenced in subclinically hypothyroid patients, but it was not influenced in subclinical hyperthyroid patients. They also reported LT4 treatment in subclinically hypothyroid patients could prevent abnormalities involving GH/IGF-1 axis.

In this study significantly lower serum IGF-1 levels were detected in total HT group in comparison to total HrT and control groups. Serum IGF-1 levels in HT subgroup were significantly lower in comparison to control group; serum IGF-1 levels in SCHT subgroup were significantly lower in comparison to control group and SCHrT subgroup. A negative correlation between TSH and IGF-1were also observed in HT subgroup. These results were compatible with that of Akin et al. [24], Schmid et al. [21] l, and Kursunluoglu et al. [23]

In this study, no correlation between resistin and IGF-1 levels was found. The reason may be limited number and age diversity of individuals in study groups. A comparement can not be made about that result because there is no study about correlation between resistin and IGF-1 in thyroid dysfunction in the literature.

In conclusion, increased resistin levels are directly related to thyroid dysfunction, and changes in levels of thyroid hormones may affect synthesis and/or secretion of resistin in adipose tissue and/or macrophages. In addition, serum IGF-1 levels decrease in hypothyroid status and correlate negatively with TSH levels. GH/IGF-1 axis may be influenced in clinical or subclinically hypothyroid patients. However, it was concluded that demographic characteristics, therapeutic variations and underlying reasons of thyroid dysfunction should be taken into consideration while interpreting the resistin and IGF-1 levels in thyroid dysfunction.

## Figures and Tables

**Table 1:** Comparisons of laboratory findings and demographic characteristics of total HT, total HrT, and healthy control groups.

Parameters	Reference ranges	Total hypothyroid group (*n* = 31) Mean ± SD Median (min-max)	Total hyperthyroid group (*n* = 30) Mean ± SD Median (min-max)	Control group (*n* = 17) Mean ± SD Median (min-max)
Age (year)	(—)	47.84 ± 13.31*	43.43 ± 15.66 39.5 (22–79)	37 ± 12.53 34 (22–72)
T-Chol (mg/dL)	120–200	235.13 ± 56.99^∗†^	162.57 ± 42.04	178.41 ± 31.64
HDL-Chol (mg/dL)	35–70	56.61 ± 13.39	52.17 ± 11.61	59.65 ± 14.11
LDL-Kol (mg/dL)	<130	144.19 ± 48.6^∗†^	89.9 ± 32.32	100.24 ± 26.88
AST (U/L)	0–50	41.58 ± 46.12^‡^ 24 (16–213)	23.43 ± 7.72	19.06 ± 4.46 18 (15–34)
ALT (U/L)	0–50	43.68 ± 58.49 21 (5–265)	24.47 ± 10.18	19.12 ± 9.76 17 (7–50)
TSH (*µ*IU/mL)	0.34–5.6	50.85 ± 75.03^∗†^ 15.74 (5.63–306.3)	0.089 ± 0.090^*α*^ 0.04 (0.015–0.279)	2.10 ± 1.26
fT4 (ng/dL)	0.58–1.64	0.53 ± 0.23*	2.08 ± 1.20^†*α*^ 1.68 (0.68–4.85)	0.83 ± 0.10 0.81 (0.73–1.03)
Resistin (ng/mL)	(—)	12.66 ± 6.04^∞^ 11.98 (5.28–30.32)	12.19 ± 7.13 11.1 (3.84–33.5)	8.45 ± 2.90
IGF-I (ng/mL)	64–345	117.22 ± 52.03^†∗^ 104 (58.8–241)	155.17 ± 51.67	184 ± 49.73

*P* < 0.05; *: hypothyroid and control; ^†^: hypothyroid and hyperthyroid; ^*α*^: hyperthyroid and control.

^‡^ *P* = 0.002 (hypothyroid and control); ^*∞*^ *P* = 0.025 (hypothyroid and control).

**Table 2:** Comparisons of laboratory findings and demographic characteristics of 4 subgroups of patients and healthy control groups.

Parameters	Hypothyroid (*n* = 15) Mean ± SD Median (min-max)	Subclinically hypothyroid (*n* = 16) Mean ± SD Median (min-max)	Hyperthyroid (*n* = 15) Mean ± SD Median (min-max)	Subclinically hyperthyroid (*n* = 15) Mean ± SD Median (min-max)	Control group (*n* = 17) Mean ± SD Median (min-max)
Age (year)	46.53 ± 13.98	49.06 ± 12.98	40.40 ± 17.36 36 (22–79)	46.47 ± 13.66	37 ± 12.53 34 (22–72)
T-Chol (mg/dL)	254.27 ± 63.78^*α*∗^	217.19 ± 44.6^*£*^	146.47 ± 33.69	178.67 ± 44.4^$^	178.41 ± 31.64
HDL-Chol (mg/dL)	56.53 ± 11.75	56.69 ± 15.16	51.53 ± 14.0	52.8 ± 9.06	59.65 ± 14.11
LDL-Chol (mg/dL)	163.9 ± 53.91^*α*$∗^	125.69 ± 35.42^*£*^	78.27 ± 22.37	101.53 ± 37.06	100.24 ± 26.88
AST (U/L)	51.6 ± 58.31* 26 (16–213)	32.19 ± 29.76 22.5 (16–136)	23.2 ± 7.8	23.67 ± 7.9 20 (16–40)	19.06 ± 4.46 18 (15–34)
ALT (U/L)	46.47 ± 56.83 22 (5–204)	41.06 ± 61.75 21 (9–265)	26.9 ± 9.6	22.0 ± 10.46	19.12 ± 9.76 17 (7–50)
TSH (*µ*IU/mL)	94.4 ± 89.97^*α*$∗^ 65.19 (9.410–306.3)	10.02 ± 5.31^*£*^ 7.67 (5.63–24.96)	0.05 ± 0.06^*≠*^ 0.02 (0.015–0.22)	0.13 ± 0.10^€^ 0.1 (0.015–0.270)	2.10 ± 1.26
fT4 (ng/dL)	0.33 ± 0.14^*α*$∗^	0.73 ± 0.08^*£*^	3.08 ± 0.88^*≠*^	1.07 ± 0.28	0.83 ± 0.10 0.81 (0.73–1.03)
Resistin (ng/mL)	13.6 ± 6.25	11.76 ± 5.89 11.35 (5.72–30.32)	9.51 ± 5.48 8.66 (4.1–24.76)	14.88 ± 7.73^#^	8.45 ± 2.90
IGF-I (ng/mL)	123.7 ± 44.03^∗†^	111.11 ± 59.35^€^ 85.95 (58.8–241)	143.73 ± 54.39	166.6 ± 47.87 148 (114–243)	184 ± 49.73

*P* < 0.05; ^#^: subclinically hyperthyroid and control; *: hypothyroid and control; ^€^: subclinically hyperthyroid and subclinically hypothyroid; ^†^: subclinically hypothyroid and control; ^*α*^: hyperthyroid and hypothyroid; ^*£*^: hyperthyroid and subclinically hypothyroid; ^$^: hypothyroid and subclinically hyperthyroid; ^*≠*^: hyperthyroid and control.

**Table 3:** Correlations between resistin, IGF-1, and TSH in total HT and total HrT groups and HT-HrT subgroups.

		IGF-1	TSH
Total hyperthyroid group (*n* = 30)	Resistin	*r* = 0.007 *P* = 0.971	***r *** = **0.463** ***P *** = **0.010**
	IGF-I	— —	*r* = −0.069 *P* = 0.718

Total hypothyroid group (*n* = 31)	Resistin	*r* = −0.026 *P* = 0.889	*r* = 0.182 *P* = 0.328
	IGF-1	— —	*r* = 0.081 *P* = 0.663

Hypothyroid subgroup (*n* = 15)	Resistin	*r* = −0.439 *P* = 0.101	*r* = 0.332 *P* = 0.226
	IGF-I	— —	***r *** = −**0.704** ***P *** = **0.003**

Hyperthyroid subgroup (*n* = 15)	Resistin	*r* = −0.086 *P* = 0.761	*r* = 0.466 *P* = 0.080
	IGF-I	— —	*r* = −0.131 *P* = 0.641

Subclinically hypothyroid subgroup	Resistin	*r* = 0.276 *P* = 0.300	*r* = 0.265 *P* = 0.322
	IGF-I	— —	*r* = 0.165 *P* = 0.542

Subclinically hyperthyroid subgroup	Resistin	*r* = −0.154 *P* = 0.583	*r* = 0.034 *P* = 0.904
	IGF-I	— —	*r* = −0.085 *P* = 0.764

**Table 4:** Within and between assay variations of resistin and IGF-1 measurements.

	Concentration	SD	CV
Resistin (ng/mL)			

Within assay	12.57	0.47	3.73
Between assay	15.32	1.07	6.97

IGF-1 (ng/mL)			

Within assay	169	6.5	3.8
Between assay	169	9.1	5.4
